# Human T-cell leukaemia virus type 1: parasitism and pathogenesis

**DOI:** 10.1098/rstb.2016.0272

**Published:** 2017-09-11

**Authors:** Charles R. M. Bangham, Masao Matsuoka

**Affiliations:** 1 Division of Infectious Diseases, Faculty of Medicine, Imperial College London, London W2 1PG, UK; 2 Department of Hematology, Rheumatology, and Infectious Diseases, Kumamoto University Faculty of Life Sciences, 1-1-1 Honjo, Kumamoto 860-8556, Japan; 3 Institute for Frontier Life and Medical Sciences, Kyoto University, 53 Shogoin Kawahara-cho, Sakyo-ku, Kyoto 606-8507, Japan

**Keywords:** HTLV-1, Tax, HBZ

## Abstract

Human T-cell leukaemia virus type 1 (HTLV-1) causes not only adult T-cell leukaemia-lymphoma (ATL), but also inflammatory diseases including HTLV-1-associated myelopathy/tropical spastic paraparesis. HTLV-1 transmits primarily through cell-to-cell contact, and generates abundant infected cells in the host in order to survive and transmit to a new host. The resulting high proviral load is closely associated with the development of ATL and inflammatory diseases. To increase the number of infected cells, HTLV-1 changes the immunophenotype of infected cells, induces proliferation and inhibits apoptosis through the cooperative actions of two viral genes, *tax* and *HTLV-1 bZIP factor* (*HBZ*). As a result, infected cells survive, proliferate and infiltrate into the tissues, which is critical for transmission of the virus. Thus, the strategy of this virus is indivisibly linked with its pathogenesis, providing a clue for prevention and treatment of HTLV-1-induced diseases.

This article is part of the themed issue ‘Human oncogenic viruses’.

## 1. Introduction

Human T-cell leukaemia virus type 1 (HTLV-1) has a long history in the human, after interspecies transmission from monkeys. HTLV-1 has a simian origin called simian T-cell leukaemia virus type 1 (STLV-1). STLV-1 is endemic in many species of Old-World monkeys and apes. The precise origins of the ancestors of HTLV-1 now present in humans remain uncertain, i.e. the species of monkey and the time and place of the putative interspecies transmission. However, it is thought to be an ancient interspecies transfer [1]. Moreover, interspecies transmission from monkeys and apes infected by STLV-1 strains are continuing, at least in Central Africa, as reported in [2]. In this review, we show how HTLV-1 transmits and survives *in vivo*, and how the strategy of the virus is associated with the pathogenesis of malignant and inflammatory diseases, with a special focus on adult T-cell leukaemia-lymphoma (ATL).

## 2. The strategy of human T-cell leukaemia virus type 1

One of the most striking characteristics of HTLV-1 is that this virus can transmit only through cell-to-cell contact [3]. Free virus is not detected in infected individuals, and free virions show very poor infectivity *in vitro*. Thus, transmission of this virus needs living infected cells. Infected cells transmit to new hosts through three routes: breast-feeding, sexual intercourse, and the parenteral route [4]. Therefore, if HTLV-1 can increase the number of infected cells in the host, it would augment transmission of this virus. Indeed, HTLV-1 generates a high frequency of infected cells *in vivo*. For this purpose, HTLV-1-infected cells need to evade host immune surveillance, promote proliferation and inhibit apoptosis. Another important issue for transmission is that infected cells enter into breast milk and semen for transmission. HTLV-1 has therefore evolved mechanisms to increase the migratory capacity of infected T cells. These two broad viral strategies—host cell proliferation and cellular motility—are closely linked to the pathogenesis of the diseases caused by this virus [5,6].

## 3. Genetic structure and gene products of human T-cell leukaemia virus type 1

**Figure 1.**
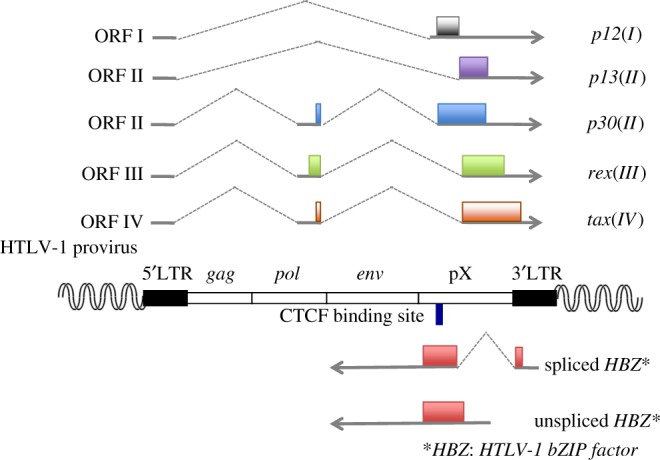
Structure of HTLV-1 provirus and its encoded genes. HTLV-1 provirus contains *gag*, *pol*, and *env* genes that encode structural proteins. In addition, *tax*, *rex*, *p12*, *p13* and *p30* are transcribed from the plus strand of the provirus. *HTLV-1 bZIP factor* (*HBZ*) is transcribed as antisense transcripts. The blue bar in the HBZ coding region shows the CTCF binding site.

HTLV-1 has a similar structure to other complex retroviruses. In addition to structural genes (*gag*, *pol* and *env*), it encodes regulatory genes (*tax* and *rex*) and accessory genes (*p12*, *p13*, *p30* and *HTLV-1 bZIP factor* (*HBZ*)) [7]. As shown in figure 1, only *HBZ* is encoded on the minus strand of the provirus and transcribed from the 3′ long terminal repeat (LTR). Other viral genes are transcribed as sense transcripts from the 5′LTR. Transcription from the 5′LTR is highly inducible by Tax, in which CREB and p300/CBP are involved [8]. Whereas *tax* is intermittently transcribed *in vivo*, *HBZ* undergoes constant expression, in which SP1 is critically involved [9]. JunD augments *HBZ* transcription by cooperating with Sp1 [10]. Tax expression is enhanced by removal of CD8^+^ T cells *in vitro*, suggesting that cytotoxic T lymphocytes (CTLs) suppress Tax expression *in vivo* [11]. These different modes of transcription may be linked with the immunogenicity of these proteins. Tax is a highly immunogenic protein, whereas the immunogenicity of HBZ protein is low [12–15]. Therefore, HTLV-1-infected cells can express HBZ under immunosurveillance of the host whereas Tax expression is very restricted.

The *tax* and *HBZ* genes are encoded respectively by the plus and minus strands of the provirus. Transcription of these genes appears to be reciprocally controlled. In valproate-treated infected cells with high Tax expression, the *HBZ* transcript was suppressed [16]. However, it is thought that these viral genes cooperate in viral replication and in proliferation of infected cells.

## 4. Infection of a new individual: routes of infection

**Figure 2.**
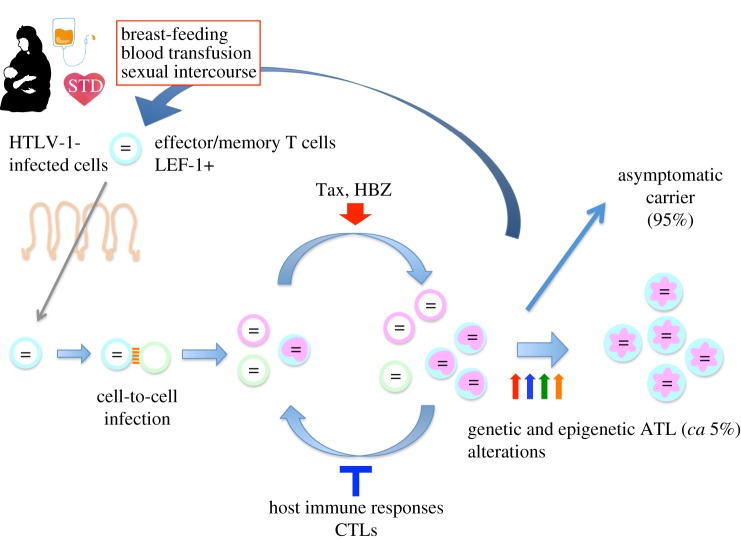
Natural history of HTLV-1 infection. HTLV-1 transmits via three different routes: (i) breast-feeding, (ii) sexual intercourse, and (iii) the parenteral route. After infection, the virus increases the number of infected cells *in vivo* through the actions of Tax and HBZ. The host immune response suppresses HTLV-1-infected cells, mainly through lysis by virus-specific cytotoxic T lymphocytes (CTLs). HTLV-1-infected cells possess the immunophenotype of effector/memory T cells, which migrate into breast milk and semen; these infected cells can transfer infection to the new host. Between 5% and 10% of HTLV-1-infected individuals develop ATL or inflammatory diseases. STD, sexually transmitted disease.

As noted above, the infectivity of free HTLV-1 virions is very poor, and HTLV-1 can transmit efficiently only through cell-to-cell infection [17]. Infected cells form a virological synapse, allowing efficient transfer of viral particles to uninfected cells, and leading to *de novo* infection [3]. Therefore, the routes of infection are limited to the following three: (i) mother-to-child, mainly via breast-feeding, (ii) sexual transmission, and (iii) blood transfusion or parenteral transmission (figure 2) [7]. In all three routes, transfer of living infected cells is essential. For transfer of infection through breast milk, it remains unknown how infected cells pass through the alimentary tract in the new host. It remains an open question whether breast-duct epithelial cells contribute to HTLV-1 transmission in the breast milk [18,19]. The HTLV-1 provirus is found mainly in effector/memory CD4^+^ T cells, indicating that this subpopulation is infected with HTLV-1 [20]. Most T cells present in breast milk and semen are effector/memory T cells [21]. Most HBZ-expressing T cells in *HBZ* transgenic mice possessed the immunophenotype of effector/memory T cells, whereas effector/memory T cells were not increased in *tax*-transgenic mice [22]. Thus, HBZ changes the immunophenotype of infected T cells, which facilitates transmission of this virus.

## 5. Spread of infection

Because primary infection with HTLV-1 is asymptomatic, there are few data on the rate of propagation of the virus during the establishment of the proviral load. In three recipients of organ transplants from an infected donor, the proviral load in the circulation doubled approximately every 1.4 days during the first few weeks of infection [23]. It is not known whether the transient immunosuppressive treatment associated with transplantation accelerated or decreased the rate of viral spread in these recipients.

Like other replication-competent exogenous retroviruses, HTLV-1 can propagate by two routes [24]. First, the integrated provirus is re-expressed, forming enveloped viral particles, which infect a new cell in which the viral genome is reverse-transcribed and the resulting double-stranded DNA is integrated into the host genome. This may be called the infectious route of replication. HTLV-1 has lost the need to release cell-free virions from the infected cell: instead, HTLV-1 spreads almost exclusively by cell-to-cell contact via a specialized structure called the virological synapse [3]. The cellular receptors for HTLV-1 are neuropilin-1 [25] and the glucose transporter GLUT-1 [26]; heparan sulfate proteoglycans also increase the efficiency of HTLV-1 infection [27]. Intercellular transfer of virus at the virological synapse may occur in pockets isolated between the two plasma membranes [28] or at the periphery of the synapse [29]; transfer via cellular conduits has also been proposed [30].

Second, mitosis of an HTLV-1-infected cell produces two daughter cells that carry the provirus at the same genomic site. In contrast to the infectious route of spread described above, this ‘mitotic’ route involves replication of the provirus by DNA Pol2, whose nucleotide misincorporation rate is about 10^5^-fold lower than that of reverse transcriptase. Mitotic replication therefore generates much less sequence diversity than infectious replication.

Integration of the HTLV-1 provirus in the host genome is not random, but is determined by factors at four successive physical scales [31]. First, integration predominates in open, transcriptionally-active chromatin. Second, integration is favoured within 100 nucleotides of genomic sites that are bound by certain proteins either directly (STAT1, TP53) or indirectly (HDAC6, Brg1) [32]. Third, the ubiquitous enzyme protein phosphatase 2A (PP2A) binds the complex of HTLV-1 integrase and the viral DNA, and influences the selection of genomic integration sites [33]. It is likely that, as in other retroviruses such as HIV-1, certain other host proteins can also bind the pre-integration complex and influence integration site selection. Fourth, retroviral integration is targeted to a primary DNA sequence motif. This DNA motif has been believed to be palindromic, but there is recent evidence that the retroviral intasome recognizes a non-palindromic motif [34]; the presence of this motif in approximately equal numbers on both the plus and the minus strands of the host genome generates a consensus sequence that is palindromic. Most naturally-infected T cell clones carry a single copy of the provirus integrated into the genome [35]; it is unknown what restricts superinfection of cells with HTLV-1.

HTLV-1 infection drives proliferation of the infected cell, through the products of the HTLV-1 genes *tax* and *HBZ* (see below), and certain HTLV-1-infected T-cell clones reach a very high abundance in the circulation. Until recently, it was believed that a typical HTLV-1-infected host carried about 100 infected T-cell clones [36], and that the observed oligoclonal proliferation both maintained persistence of the virus *in vivo* and contributed to the pathogenesis of the inflammatory and malignant diseases associated with HTLV-1. Recent evidence from high-throughput sequencing and quantification of HTLV-1 integration sites has changed this view: the number of HTLV-1-positive T-cell clones in the circulation in each host is of the order of 10^4^–10^5^ [31], and it is the clonal diversity, not the degree of oligoclonal proliferation, that correlates with the proviral load [37]. These observations imply that high clonal diversity, rather than oligoclonal proliferation, predisposes to the inflammatory and malignant diseases caused by HTLV-1.

Both CD4^+^ and CD8^+^ T cells are infected by HTLV-1: about 95% of the proviral load is present in CD4^+^ T cells, and 5% in CD8^+^ T cells [38]. Both CD8^+^ and CD4^+^ T cells are preferentially infected with the virus if they are HTLV-1-antigen-specific [11,39]. Most cell types can be infected with HTLV-1 *in vitro*; small numbers of dendritic cells, monocyte/macrophages and epithelial cells are infected *in vivo*. It has been reported that infected dendritic cells and macrophages play important roles in propagation of HTLV-1 [40].

## 6. Human T-cell leukaemia virus type 2

Human T-cell leukaemia virus type 2 (HTLV-2) was discovered in a T-cell line established from a patient with T-cell variant hairy cell leukaemia [41]. However, an association between HTLV-2 and leukaemia has not been established, and it is now thought that HTLV-2 does not cause leukaemia. HTLV-2 encodes Tax-2, whose sequence is similar to that of HTLV-1 Tax. In addition, HTLV-2 encodes the antisense protein of HTLV-2 (APH-2) on the minus strand of the provirus [42]. However, unlike HBZ, APH-2 does not have growth-promoting activity [43]. HTLV-2 almost exclusively infects CD8^+^ T cells *in vivo* [38], whereas HTLV-1 is present mainly in CD4^+^ T cells. However, the precise mechanisms of cell specificity of HTLV-1 and HTLV-2 remain to be elucidated.

## 7. Immune response to human T-cell leukaemia virus type 1

The immune response to HTLV-1 has been extensively reviewed elsewhere [44–46], and will be summarized briefly here.

### (a) Host restriction factors

Efficient spread of HTLV-1 virions across the intimate cell–cell contact in the virological synapse minimizes the ability of tetherin to inhibit HTLV-1 propagation [47]. The deaminase APOBEC3G, which plays an important role in restricting lentiviral infections, also has activity against HTLV-1. APOBEC3G can generate nonsense mutations *in vivo*, but since it targets the minus strand during reverse transcription, it spares *HBZ* [48]; the consequent silencing of plus-strand gene products and sustained *HBZ* expression may favour viral persistence. Like HIV-1, HTLV-1 incorporates APOBEC3G into the virion, but this incorporation is limited by a peptide motif in the nucleocapsid [49].

SAMHD1, which limits the supply of nucleoside triphosphates for virion replication, inhibits HTLV-1 infection of macrophages [50]. Type 1 interferon has activity against HTLV-1, but the virus suppresses Type 1 interferon production both by inducing the cytokine suppressor SOCS1 [51] and by Tax protein-mediated inhibition of RIG-I and MDA5 [52]. However, HTLV-1 Tax strongly induces interferon-γ production by the infected cell [53], and chronic stimulation of interferon response genes is associated with the inflammatory disease HAM/TSP [54].

### (b) Lymphocyte response to human T-cell leukaemia virus type 1

The frequency and activity of NK and NKT-like cells are abnormally low in HAM/TSP [55–57], but the significance of this reproducible observation is not understood. The HLA Class 1-restricted CD8^+^ CTL plays a dominant role in host protection in most viral infections, and the CTL response is an important determinant of the outcome of HTLV-1 infection. HTLV-1-infected individuals typically have a high frequency of persistently activated, HTLV-1-specific CTLs in the circulation; the activated state indicates recent exposure to newly synthesized viral antigen, and therefore demonstrates that the virus is persistently expressed *in vivo*. Although the Tax protein is highly immunodominant in the anti-HTLV-1 CTL response, it is the efficacy or ‘quality’ of the CTL response to the HBZ protein that is associated with control of the virus *in vivo* [14]. The class 1 HLA genotype of the host, which determines the antigen specificity and quality of the CTL response, therefore determines the proviral load and the risk of HAM/TSP [44]; in southern Japan, HLA-A*02 and Cw*08 are associated with protection against HAM/TSP, whereas HLA-B*54 is associated with a higher risk of the disease [58]. For reasons that are not yet understood, the killer immunoglobulin-like receptor KIR2DL2 enhances both the protective (HLA-A*02) and the pathogenic (HLA-B*54) HLA Class 1-associated effects in HTLV-1 infection [59].

The frequency of HTLV-1-specific CD4^+^ T cells is significantly higher in patients with HAM/TSP than in asymptomatic carriers of the virus. CD4^+^ T cells predominate in the early, active lesions in the central nervous system in HAM/TSP [60], and the predominantly Th1 response is likely to contribute to the pathogenesis of HAM/TSP [54,61].

HTLV-1 Tax protein induces the infected CD4^+^ T cell to secrete the chemokine CCL22, which maintains a high frequency of CD4^+^ Foxp3^+^ cells in the circulation because these cells characteristically express the CCL22 receptor, CCR4 [62]. The regulatory phenotype of the Foxp3^+^ cells may diminish the protective effect of the anti-HTLV-1 CTL response [63]. Although adult T-cell leukaemia cells frequently express Foxp3, ATL is not necessarily a tumour of regulatory T cells [64,65].

## 8. Mechanisms of viral persistence in the chronic phase of infection

### (a) Roles of HBZ and Tax in maintaining clonal longevity and cell turnover

After infection, HTLV-1 increases the proviral load mainly by driving proliferation of infected cells (mitotic division) [66,67]. Indeed, inhibitors of reverse transcriptase or integrase did not change the proviral load in HTLV-1-infected individuals, indicating that *de nov*o infection does not contribute significantly to the proviral load in the chronic phase [68]. HBZ and Tax play critical roles to maintain clonal longevity. HBZ perturbs the localization and function of FoxO3a, a critical transcriptional activator of the genes encoding Bim and also Fas ligand, which results in inhibited apoptosis [69]. HBZ also interacts with the Rb/E2F-1 complex and promotes cell cycle progression [70]. Furthermore, HBZ determines the immunophenotype of infected cells, including ATL cells: HBZ induces expression on the cell surface of TIGIT and CCR4 [71,72], which are implicated in the infiltration and proliferation of HTLV-1-infected cells.

In addition to these functions of HBZ protein, *HBZ* RNA possesses functions that are distinct from those of its protein. *HBZ* RNA is more strongly retained in the nucleus than the other viral gene transcripts. *HBZ* RNA inhibits apoptosis, and promotes proliferation of expressing cells [73,74]. These mechanisms could reduce production of HBZ protein, which is recognized by the host immune system.

Tax strongly activates NFκB, which leads to expression of the anti-apoptotic gene *c-FLIP* [75,76] and genes associated with cell cycling including cyclin D2, cyclin E, E2F1, CDK2, CDK4 and CDK6 [77]. In addition, Tax promotes cell proliferation and cell cycling through activation of the PI3 K/Akt pathway [78]. Thus, Tax also inhibits apoptosis and promotes cell proliferation. The function of Tax is impaired by transcription factors in the classical Wnt pathway, TCF-1/LEF-1, in the thymus [79]. However, expression of TCF-1/LEF-1 is suppressed in effector/memory T cells, indicating that Tax can function in these cells, so enabling HTLV-1 to persist in peripheral effector/memory T cells *in vivo*.

### (b) Regulation of proviral latency *in vivo*

The *HBZ* gene is persistently expressed at a low level in most—if not all—infected cells *in vivo* [73]. The plus-strand products are usually undetectable in freshly isolated peripheral blood mononuclear cells (PBMCs) but, as noted above, the persistently activated CTL response to Tax, Pol and Gag indicates that these antigens are frequently expressed *in vivo*. It is unknown what regulates this differential expression of plus and minus strands and, in particular, the frequency, intensity and duration of what are presumably bursts of plus-strand expression.

Taniguchi *et al.* [80] showed that DNA methylation might explain the proviral silencing *in vivo*, but they also observed an unexplained border in the methylation in the pX region of the provirus. It has now been shown [81] that CTCF, the key chromatin architectural protein and insulator-binding protein, binds to the provirus at its epigenetic border, at which the pattern of several other epigenetic marks also changes. It is possible that CTCF binding, which may be reversible, allows temporary silencing of plus-strand expression at the same time as persistent negative-strand (*HBZ*) expression. Another major function of CTCF is to form chromatin loops, both to organize the chromatin structure and to bring enhancers near their cognate promoters. The abnormal CTCF-binding site present in the HTLV-1 provirus can indeed form loops with flanking host chromatin [81]; this looping may contribute to leukaemogenesis by allowing LTR-mediated activation of host genes (see below).

The genomic integration site influences the expression of the provirus, and consequently each infected T-cell clone will have its own characteristics of proviral expression. Two chief specific features of the integration site are associated with the regulation of plus-strand expression [32]. First, proximity (within 100 base pairs) to certain transcription factors that bind to chromatin either directly (STAT1, P53) or indirectly (HDAC6, BRG1). The mechanistic explanation for these observations is not yet known. Second, the orientation of the provirus relative to the transcriptional orientation of the nearest host gene. The presence of a host promoter in the same transcriptional sense upstream of the 5′LTR is associated with transcriptional silencing of the HTLV-1 plus strand, whereas a host promoter upstream of the 3′LTR (in the same sense as the proviral negative strand) is associated with activation of plus-strand expression. These observations suggest that transcriptional interference plays a part in regulating HTLV-1 proviral latency.

Two central questions remain in the regulation of HTLV-1 proviral latency. First, what causes (or allows) the rapid spontaneous transcriptional activation of the provirus when PBMCs are taken *ex vivo*? The answer to this question will illuminate the mechanisms that maintain plus-strand latency *in vivo*. Second, what are the molecular mechanisms that give rise to cell-to-cell heterogeneity in proviral expression, and what is the importance of this heterogeneity in viral persistence?

### (c) Structure and dynamics of human T-cell leukaemia virus type 1 clonality *in vivo*

It was formerly believed that a typical host possessed about 100 clones of HTLV-1-infected T cells [36], and that the most abundant (‘oligoclonally expanded’) clones accounted for the Tax expression, the high proviral load and the HTLV-1-associated diseases [82,83]. However, quantitative high-throughput analysis has revealed a very different picture. The number of clones carried by each host is usually between 10^4^ and 10^5^ (ranging from 10^3^ to 10^6^). It is the large number of low-abundance clones that constitute the high proviral load [37]: these clones frequently express Tax, and turn over rapidly *in vivo*.

**Figure 3.**
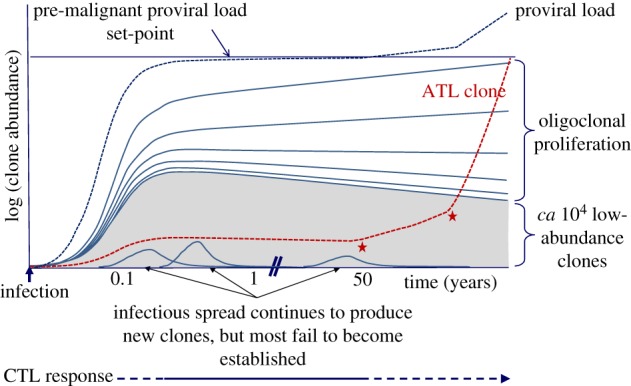
Schematic depiction of HTLV-1 clonality over the course of infection. Each line on the figure represents the growth of one HTLV-1^+^ T-cell clone; the grey shaded area represents a large number of lower-abundance clones. The red dashed line shows the growth trajectory of a clone that undergoes malignant transformation after 50 years; the red asterisks denote the acquisition of tumour driver mutations; in ATL these are frequent in certain signalling pathways: T-cell receptor; NFκB; CCR4; p53; and Notch-1 [84–87]. The pre-malignant clone is shown to originate in early infection; this is likely [31,88], but not necessary. During the first few weeks of infection, before the emergence of an effective cytotoxic T-lymphocyte response, the virus spreads rapidly by cell-to-cell contact through the virological synapse; the number of clones of infected T lymphocytes typically rises to 10^4^ to 10^5^ when the proviral load set-point is reached, after approximately one to two months [23,89]. In the chronic phase of infection, CTLs restrict this infectious mode of spread [44], and the proviral load is maintained by continued proliferation of existing clones. In this phase, there is a quasi-equilibrium between viral propagation and the host immune response; while the proviral load remains approximately constant, the abundant clones grow in abundance and the low-abundance clones shrink, leading to a progressive rise in the oligoclonality index [37]. The abundant clones appear to last for the lifetime of the host [37]. During chronic infection the abundant, persistently activated anti-Tax CTL response demonstrates that Tax expression is frequent *in vivo* [45]. Since Tax expression is normally undetectable in fresh PBMCs, we infer that Tax expression is intermittent *in vivo*. Virus-specific CTLs may persist during active ATL; it remains to be tested whether boosting the CTL response can be used as an adjunct to therapy. Constant cell division leads to the accumulation of replicative mutations, which increase the probability of malignant transformation [90]. ATL usually arises after 4–6 decades of infection, and so is more frequent in individuals infected during childhood. The risk of ATL may also be correlated with the proviral load, which in turn is correlated with the number of HTLV-1-infected T-cell clones, not with the degree of oligoclonality [37].

The current picture of HTLV-1 clonality is depicted schematically in figure 3. We postulate that constant pressure exerted by the host immune response limits infectious spread during the chronic phase of infection, and selects for persistence of clones with an optimal pattern of proviral expression. This optimal pattern consists of minimal but persistent HBZ expression, and bursts of plus-strand expression that may be driven by cellular stress, such as when lymphocytes are transferred between individuals, to promote viral transmission. The CTL response to the HBZ protein limits the proviral load; the virus minimizes the effect of this force by restricting the expression and the translation of HBZ mRNA and the immunogenicity of the HBZ protein. Once the proviral load set-point (quasi-equilibrium) is reached, the rate of establishment of new long-lived clones is likely to be restricted by the CTL response to HBZ, Tax and other viral antigens, and by competition for resources with pre-existing clones.

## 9. Mechanisms of oncogenesis

For many years, the Tax protein was believed to be necessary and sufficient to cause malignant transformation of HTLV-1-infected cells. Transduction of Tax-expressing vectors can immortalize T cells *in vitro* [91,92], and transgenic expression of Tax induced cancers *in vivo*. However, the cell type of the cancer caused by Tax depended on the promoter. Tax expression by the granzyme B promoter induced a tumour of NK cells [93], whereas pX expression driven by the H-2Kd promoter caused breast cancers [94]. Thus, these findings indicate that high or persistent expression of Tax is oncogenic. However, several more recent lines of evidence suggest that, in natural HTLV-1 infection, HBZ rather than Tax is the critical element in HTLV-1 oncogenesis.

The most direct evidence of HTLV-1 infection in ATL cells is the presence of the monoclonally integrated provirus. Therefore, analysis of the structure and genomic integration site of HTLV-1 proviruses in ATL cells can provide critical clues on leukaemogenesis by HTLV-1. Studies of HTLV-1 proviruses and transcripts of viral genes showed that ATL cells do not express Tax in approximately half the cases of ATL. There are three known mechanisms to inactivate Tax expression: (i) nonsense mutations of the *tax* gene, (ii) DNA methylation of the 5′LTR, and (iii) deletion of the 5′LTR. However, HBZ is expressed in all ATL cases, suggesting that HBZ is indispensable. DNA methylation of the 5′LTR accumulates during the natural course of infection, which finally silences transcription of the sense strand from the 5′LTR [95]. However, DNA methylation does not extend to the pX region and the 3′LTR [80], which is critical for *HBZ* transcription. Recently, a CTCF-binding region was found in the pX region, which may account for the arrest of DNA methylation before pX and 3′LTR, and ensure continued HBZ expression [81]. Furthermore, the malignant clone in some cases of ATL contains a defective provirus that lacks the 5′LTR (type 2 defective provirus) which is generated before genomic integration of the provirus [96]. The lack of the 5′LTR precludes expression of Tax in some cases.

Nonsense mutations of the *tax* gene were found in approximately 10% of ATL cases. There is a hotspot of nonsense mutations in the HTLV-1 provirus in a target sequence of APOBEC3G [48], suggesting that these nonsense mutations are generated by APOBEC3G during reverse transcription. Furthermore, this nonsense mutation of the *tax* gene was found in some asymptomatic carriers [48]. These findings demonstrate that nonsense mutations are generated before infection, and HTLV-1-infected cells carrying a nonsense mutation in the *tax* gene can transform to ATL cells, suggesting that HBZ plays critical roles in oncogenesis.

**Figure 4.**
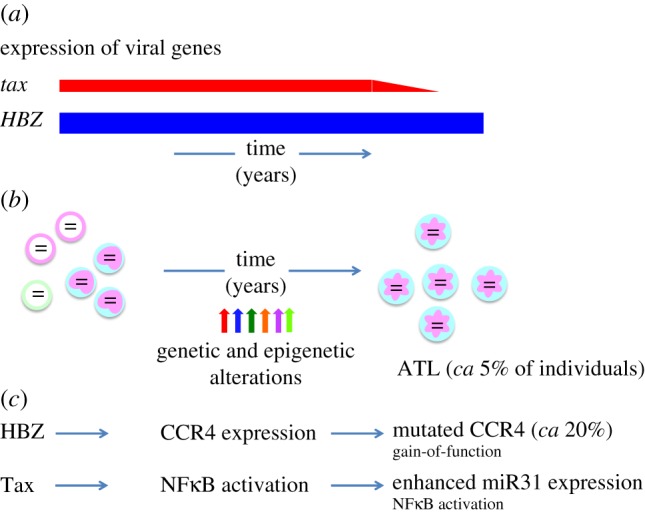
Leukaemogenesis by viral genes, genetic and epigenetic alterations. About 5% of HTLV-1-infected individuals develop ATL after a long latent period. (*a*) Tax and HBZ play critical roles in leukaemogenesis by HTLV-1. *HBZ* is constantly expressed while the *tax* gene is sporadically transcribed. Tax and HBZ modulate the immunophenotype of ATL cells, inhibit apoptosis and promote proliferation. *HBZ* is expressed in all ATL cases while *tax* is not expressed in approximately half of ATL cases. (*b*) Subsequent genetic and epigenetic alterations that accumulate during the long lifetime of the infected clone fix or potentiate these phenotypic and functional changes. (*c*) For example, HBZ induces CCR4 expression, which leads to increased migration and proliferation of infected cells. Gain-of-function mutations of CCR4 were found in approximately 20% of ATL cases. Similarly, Tax strongly activates NFκB. Increased miR31 expression leads to NFκB activation in the absence of Tax.

A long latent period is necessary before onset of ATL, suggesting that multiple genetic and epigenetic alterations are needed for ATL (figure 4). Recently, extensive studies of genomes in ATL cells revealed the landscape of genetic and epigenetic changes [84]. Interestingly, genetic alterations accumulated in the genes associated with pathways that Tax and HBZ target. These findings suggest the following scenario. HTLV-1-infected clones persist and proliferate *in vivo* through the actions of HBZ and Tax during asymptomatic carriage of the virus. Thereafter, genetic and epigenetic alterations fix or potentiate these changes. For example, HBZ induces CCR4 expression [72]. Gain-of-function mutations of the CCR4 gene are associated with proliferation and infiltration of ATL cells (figure 4) [84,85]. Furthermore, Tax strongly activates the NFκB pathway. Expression of miR31 enhances NFκB in ATL cells even in the absence of Tax [97]. These targets of viral proteins and mutations are important for therapy in ATL.

## 10. Strategies to prevent and treat adult T-cell leukaemia-lymphoma

### (a) Current approaches to management of adult T-cell leukaemia-lymphoma

There are four subtypes of ATL according to clinical criteria: acute, lymphoma-type, chronic and smouldering types [98]. In general, patients with acute, lymphoma-type or unfavourable chronic type ATL are treated, whereas patients with typical chronic or smouldering ATL are carefully observed. ATL patients are usually treated with combination chemotherapy including VCAP-AMP-VECP (also known as LSG15) therapy, or CHOP therapy. However, the prognosis of these patients remains poor despite intensive chemotherapy. On the other hand, some patients who received haematopoietic stem cell transplantation have achieved long-term survival [99]. It has been reported that anti-viral immunity was enhanced in these patients, which suggests the use of immunotherapy as an adjunct to the treatment of ATL [100].

It has been reported that sustained treatment with azidothymidine (AZT) plus interferon-α can be effective in cases of ATL of the leukaemic types, but is less effective in the lymphoma type [101]. Although AZT is a reverse transcriptase inhibitor, the combination of AZT with interferon-α does not impair HTLV-1 replication; this therapy acts through a mechanism other than anti-viral activity [102] that is not yet understood. Combination therapy using arsenic trioxide, interferon-α and zidovudine was reported to be effective in chronic ATL patients [103].

Most HTLV-1 infected cells and ATL cells express the chemokine receptor CCR4 on their surfaces [104]. CCR4 expression is implicated in migration and proliferation of T cells. Recently, it has been reported that HBZ induces CCR4 expression through enhanced GATA3 transcription [72]. An anti-CCR4 monoclonal antibody, mogamulizumab, has been developed for treatment of ATL patients [105]. Antibody-dependent cell-mediated cytotoxicity (ADCC) is thought to be a major mechanism by which this antibody strongly suppresses ATL *in vivo* [106]. CCR4 is also expressed on active regulatory T (Treg) cells [107]. After administration of mogamulizumab, ATL cells were suppressed by ADCC exerted by NK cells. At the same time, anti-Tax and anti-HBZ CTLs were activated in these patients, which possibly suppress proliferation of ATL cells [108]. Thus, the anti-CCR4 antibody exerts dual beneficial functions *in vivo*, which may account for the prolonged suppressive effect of this antibody on ATL cells.

### (b) Possible future approaches to management of adult T-cell leukaemia-lymphoma

As discussed above, HTLV-1 persists *in vivo* during chronic infection mainly by sustained proliferation of infected cells. The high resulting proviral load is also critical in the transmission of the virus, which requires transfer of infected cells to the new host. Therefore, a logical strategy to prevent ATL is to reduce the number of infected cells. Immune responses, primarily CTLs, strongly suppress proliferation of ATL cells and HTLV-1-infected cells. Thus, vaccines against Tax and HBZ might be effective in preventing HTLV-1-associated diseases. In addition, the recent genetic and epigenetic analyses of ATL cells reveal critical pathways and molecules for ATL. Therapy targeted to these pathways might improve the prognosis of this intractable disease.

## Data accessibility

This article has no additional data.

## References

[1] Reid MJ et al. 2016 Detailed phylogenetic analysis of primate T-lymphotropic virus type 1 (PTLV-1) sequences from orangutans (*Pongo pygmaeus*) reveals new insights into the evolutionary history of PTLV-1 in Asia. Infect. Genet. Evol. 43, 434–450. (10.1016/j.meegid.2016.05.036) 27245152 PMC11332081

[2] Filippone C, Betsem E, Tortevoye P, Cassar O, Bassot S, Froment A, Fontanet A, Gessain A 2015 A severe bite from a nonhuman primate is a major risk factor for HTLV-1 infection in hunters from Central Africa. Clin. Infect. Dis. 60, 1667–1676. (10.1093/cid/civ145) 25722199

[3] Igakura T, Stinchcombe JC, Goon PK, Taylor GP, Weber JN, Griffiths GM, Tanaka Y, Osame M, Bangham CR 2003 Spread of HTLV-I between lymphocytes by virus-induced polarization of the cytoskeleton. Science 299, 1713–1716. (10.1126/science.1080115) 12589003

[4] Matsuoka M, Jeang KT 2011 Human T-cell leukemia virus type 1 (HTLV-1) and leukemic transformation: viral infectivity, Tax, HBZ and therapy. Oncogene 30, 1379–1389. (10.1038/onc.2010.537) 21119600 PMC3413891

[5] Yamamoto-Taguchi N, Satou Y, Miyazato P, Ohshima K, Nakagawa M, Katagiri K, Kinashi T, Matsuoka M 2013 HTLV-1 bZIP factor induces inflammation through labile Foxp3 expression. PLoS Pathog. 9, e1003630 (10.1371/journal.ppat.1003630) 24068936 PMC3777874

[6] Ma G, Yasunaga J, Fan J, Yanagawa S, Matsuoka M 2013 HTLV-1 bZIP factor dysregulates the Wnt pathways to support proliferation and migration of adult T-cell leukemia cells. Oncogene 32, 4222–4230. (10.1038/onc.2012.450) 23045287

[7] Matsuoka M, Jeang KT 2007 Human T-cell leukaemia virus type 1 (HTLV-1) infectivity and cellular transformation. Nat. Rev. 7, 270–280. (10.1038/nrc2111) 17384582

[8] Nyborg JK, Egan D, Sharma N 2010 The HTLV-1 Tax protein: revealing mechanisms of transcriptional activation through histone acetylation and nucleosome disassembly. Biochim. Biophys. Acta 1799, 266–274. (10.1016/j.bbagrm.2009.09.002) 19782779

[9] Yoshida M, Satou Y, Yasunaga J, Fujisawa J, Matsuoka M 2008 Transcriptional control of spliced and unspliced human T-cell leukemia virus type 1 bZIP factor (*HBZ*) gene. J. Virol. 82, 9359–9368. (10.1128/JVI.00242-08) 18653454 PMC2546946

[10] Gazon H, Lemasson I, Polakowski N, Cesaire R, Matsuoka M, Barbeau B, Mesnard JM, Peloponese JM Jr 2012 Human T-cell leukemia virus type 1 (HTLV-1) bZIP factor requires cellular transcription factor JunD to upregulate HTLV-1 antisense transcription from the 3′ long terminal repeat. J. Virol. 86, 9070–9078. (10.1128/JVI.00661-12) 22696638 PMC3416116

[11] Hanon E et al. 2000 Abundant Tax protein expression in CD4^+^ T cells infected with human T-cell lymphotropic virus type I (HTLV-I) is prevented by cytotoxic T lymphocytes. Blood 95, 1386–1392. 10666215

[12] Jacobson S, Shida H, McFarlin DE, Fauci AS, Koenig S 1990 Circulating CD8^+^ cytotoxic T lymphocytes specific for HTLV-I pX in patients with HTLV-I associated neurological disease. Nature 348, 245–248. (10.1038/348245a0) 2146511

[13] Kannagi M et al. 1991 Predominant recognition of human T cell leukemia virus type I (HTLV-I) pX gene products by human CD8^+^ cytotoxic T cells directed against HTLV-I-infected cells. Int. Immunol. 3, 761–767. (10.1093/intimm/3.8.761) 1911545

[14] Macnamara A et al. 2010 HLA class I binding of HBZ determines outcome in HTLV-1 infection. PLoS Pathog. 6, e1001117 (10.1371/journal.ppat.1001117) 20886101 PMC2944806

[15] Hilburn S, Rowan A, Demontis MA, Macnamara A, Asquith B, Bangham CR, Taylor GP 2011 *In vivo* expression of human T-lymphotropic virus type 1 basic leucine-zipper protein generates specific CD8^+^ and CD4^+^ T-lymphocyte responses that correlate with clinical outcome. J. Infect. Dis. 203, 529–536. (10.1093/infdis/jiq078) 21208912 PMC3071236

[16] Belrose G et al. 2011 Effects of valproate on Tax and HBZ expression in HTLV-1 and HAM/TSP T lymphocytes. Blood 118, 2483–2491. (10.1182/blood-2010-11-321364) 21505188

[17] Mazurov D, Ilinskaya A, Heidecker G, Lloyd P, Derse D 2010 Quantitative comparison of HTLV-1 and HIV-1 cell-to-cell infection with new replication dependent vectors. PLoS Pathog. 6, e1000788 (10.1371/journal.ppat.1000788) 20195464 PMC2829072

[18] Southern SO, Southern PJ 1998 Persistent HTLV-I infection of breast luminal epithelial cells: a role in HTLV transmission? Virology 241, 200–214. (10.1006/viro.1997.8978) 9499795

[19] Martin-Latil S et al. 2012 Transcytosis of HTLV-1 across a tight human epithelial barrier and infection of subepithelial dendritic cells. Blood 120, 572–580. (10.1182/blood-2011-08-374637) 22589473

[20] Yasunaga J et al. 2001 Impaired production of naive T lymphocytes in human T-cell leukemia virus type I-infected individuals: its implications in the immunodeficient state. Blood 97, 3177–3183. (10.1182/blood.V97.10.3177) 11342446

[21] Bertotto A, Gerli R, Fabietti G, Crupi S, Arcangeli C, Scalise F, Vaccaro R 1990 Human breast milk T lymphocytes display the phenotype and functional characteristics of memory T cells. Eur. J. Immunol. 20, 1877–1880. (10.1002/eji.1830200838) 2120066

[22] Satou Y et al. 2011 HTLV-1 bZIP factor induces T-cell lymphoma and systemic inflammation *in vivo*. PLoS Pathog. 7, e1001274 (10.1371/journal.ppat.1001274) 21347344 PMC3037353

[23] Cook LB et al. 2016 Rapid dissemination of human T-lymphotropic virus type 1 during primary infection in transplant recipients. Retrovirology 13, 3 (10.1186/s12977-015-0236-7) 26745892 PMC4706667

[24] Overbaugh J, Bangham CR 2001 Selection forces and constraints on retroviral sequence variation. Science 292, 1106–1109. (10.1126/science.1059128) 11352065

[25] Ghez D et al. 2006 Neuropilin-1 is involved in human T-cell lymphotropic virus type 1 entry. J. Virol. 80, 6844–6854. (10.1128/JVI.02719-05) 16809290 PMC1489069

[26] Manel N, Kim FJ, Kinet S, Taylor N, Sitbon M, Battini JL 2003 The ubiquitous glucose transporter GLUT-1 is a receptor for HTLV. Cell 115, 449–459. (10.1016/S0092-8674(03)00881-X) 14622599

[27] Lambert S et al. 2009 HTLV-1 uses HSPG and neuropilin-1 for entry by molecular mimicry of VEGF165. Blood 113, 5176–5185. (10.1182/blood-2008-04-150342) 19270265 PMC2686187

[28] Majorovits E, Nejmeddine M, Tanaka Y, Taylor GP, Fuller SD, Bangham CR 2008 Human T-lymphotropic virus-1 visualized at the virological synapse by electron tomography. PLoS ONE 3, e2251 (10.1371/journal.pone.0002251) 18509526 PMC2386264

[29] Pais-Correia AM, Sachse M, Guadagnini S, Robbiati V, Lasserre R, Gessain A, Gout O, Alcover A, Thoulouze MI 2010 Biofilm-like extracellular viral assemblies mediate HTLV-1 cell-to-cell transmission at virological synapses. Nat. Med. 16, 83–89. (10.1038/nm.2065) 20023636

[30] Van Prooyen N et al. 2010 Human T-cell leukemia virus type 1 p8 protein increases cellular conduits and virus transmission. Proc. Natl Acad. Sci. USA 107, 20 738–20 743. (10.1073/pnas.1009635107) PMC2996430 21076035

[31] Bangham CR, Cook LB, Melamed A 2014 HTLV-1 clonality in adult T-cell leukaemia and non-malignant HTLV-1 infection. Semin. Cancer Biol. 26C, 89–98. (10.1016/j.semcancer.2013.11.003) PMC4062949 24316494

[32] Melamed A, Laydon DJ, Gillet NA, Tanaka Y, Taylor GP, Bangham CR 2013 Genome-wide determinants of proviral targeting, clonal abundance and expression in natural HTLV-1 infection. PLoS Pathog. 9, e1003271 (10.1371/journal.ppat.1003271) 23555266 PMC3605240

[33] Maertens GN 2016 B'-protein phosphatase 2A is a functional binding partner of delta-retroviral integrase. Nucleic Acids Res. 44, 364–376. (10.1093/nar/gkv1347) 26657642 PMC4705670

[34] Kirk PDW, Huvet M, Melamed A, Maertens GN, Bangham CRM 2016 Retroviruses integrate into a shared, non-palindromic DNA motif. Nat. Microbiol. 2, 16212 (10.1038/nmicrobiol.2016.212) 27841853 PMC7613964

[35] Cook LB, Rowan AG, Melamed A, Taylor GP, Bangham CR 2012 HTLV-1-infected T cells contain a single integrated provirus in natural infection. Blood 120, 3488–3490. (10.1182/blood-2012-07-445593) 22955925 PMC3482858

[36] Wattel E, Cavrois M, Gessain A, Wain-Hobson S 1996 Clonal expansion of infected cells: a way of life for HTLV-I. J. Acquir. Immune Defic. Syndr. Hum. Retrovirol. 13(Suppl. 1), S92–S99. (10.1097/00042560-199600001-00016) 8797710

[37] Gillet NA et al. 2011 The host genomic environment of the provirus determines the abundance of HTLV-1-infected T cell clones. Blood 117, 3113–3122. (10.1182/blood-2010-10-312926) 21228324 PMC3062313

[38] Melamed A, Laydon DJ, Al Khatib H, Rowan AG, Taylor GP, Bangham CR 2015 HTLV-1 drives vigorous clonal expansion of infected CD8^+^ T cells in natural infection. Retrovirology 12, 91 (10.1186/s12977-015-0221-1) 26552867 PMC4640420

[39] Goon PK et al. 2004 Human T cell lymphotropic virus type I (HTLV-I)-specific CD4^+^ T cells: immunodominance hierarchy and preferential infection with HTLV-I. J. Immunol. 172, 1735–1743. (10.4049/jimmunol.172.3.1735) 14734756

[40] de Castro-Amarante MF et al. 2015 Human T cell leukemia virus type 1 infection of the three monocyte subsets contributes to viral burden in humans. J. Virol. 90, 2195–2207. (10.1128/JVI.02735-15) 26608313 PMC4810698

[41] Kalyanaraman VS, Sarngadharan MG, Robert-Guroff M, Miyoshi I, Golde D, Gallo RC 1982 A new subtype of human T-cell leukemia virus (HTLV-II) associated with a T-cell variant of hairy cell leukemia. Science 218, 571–573. (10.1126/science.6981847) 6981847

[42] Halin M et al. 2009 Human T-cell leukemia virus type 2 produces a spliced antisense transcript encoding a protein that lacks a classic bZIP domain but still inhibits Tax2-mediated transcription. Blood 114, 2427–2438. (10.1182/blood-2008-09-179879) 19602711 PMC2746472

[43] Douceron E, Kaidarova Z, Miyazato P, Matsuoka M, Murphy EL, Mahieux R 2012 HTLV-2 APH-2 expression is correlated with proviral load but APH-2 does not promote lymphocytosis. J. Infect. Dis. 205, 82–86. (10.1093/infdis/jir708) 22065675 PMC3242747

[44] Bangham CR 2009 CTL quality and the control of human retroviral infections. Eur. J. Immunol. 39, 1700–1712. (10.1002/eji.200939451) 19582737

[45] Bangham CR, Osame M 2005 Cellular immune response to HTLV-1. Oncogene 24, 6035–6046. (10.1038/sj.onc.1208970) 16155610

[46] Journo C, Mahieux R 2011 HTLV-1 and innate immunity. Viruses 3, 1374–1394. (10.3390/v3081374) 21994785 PMC3185810

[47] Ilinskaya A, Derse D, Hill S, Princler G, Heidecker G 2013 Cell-cell transmission allows human T-lymphotropic virus 1 to circumvent tetherin restriction. Virology 436, 201–209. (10.1016/j.virol.2012.11.012) 23260108

[48] Fan J, Ma G, Nosaka K, Tanabe J, Satou Y, Koito A, Wain-Hobson S, Vartanian JP, Matsuoka M 2010 APOBEC3G generates nonsense mutations in human T-cell leukemia virus type 1 proviral genomes *in vivo*. J. Virol. 84, 7278–7287. (10.1128/JVI.02239-09) 20463074 PMC2898234

[49] Derse D, Hill SA, Princler G, Lloyd P, Heidecker G 2007 Resistance of human T cell leukemia virus type 1 to APOBEC3G restriction is mediated by elements in nucleocapsid. Proc. Natl Acad. Sci. USA 104, 2915–2920. (10.1073/pnas.0609444104) 17299050 PMC1815281

[50] Sze A, Belgnaoui SM, Olagnier D, Lin R, Hiscott J, van Grevenynghe J 2013 Host restriction factor SAMHD1 limits human T cell leukemia virus type 1 infection of monocytes via STING-mediated apoptosis. Cell Host Microbe 14, 422–434. (10.1016/j.chom.2013.09.009) 24139400

[51] Oliere S et al. 2010 HTLV-1 evades type I interferon antiviral signaling by inducing the suppressor of cytokine signaling 1 (SOCS1). PLoS Pathog. 6, e1001177 (10.1371/journal.ppat.1001177) 21079688 PMC2973829

[52] Hyun J, Ramos JC, Toomey N, Balachandran S, Lavorgna A, Harhaj E, Barber GN 2015 Oncogenic human T-cell lymphotropic virus type 1 Tax suppression of primary innate immune signaling pathways. J. Virol. 89, 4880–4893. (10.1128/JVI.02493-14) 25694597 PMC4403453

[53] Hanon E, Goon P, Taylor GP, Hasegawa H, Tanaka Y, Weber JN, Bangham CR 2001 High production of interferon γ but not interleukin-2 by human T-lymphotropic virus type I-infected peripheral blood mononuclear cells. Blood 98, 721–726. (10.1182/blood.V98.3.721) 11468172

[54] Tattermusch S, Skinner JA, Chaussabel D, Banchereau J, Berry MP, McNab FW, O'Garra A, Taylor GP, Bangham CRM 2012 Systems biology approaches reveal a specific IFN-inducible signature in HTLV-1 associated myelopathy. PLoS Pathog. 8, e1002480 (10.1371/journal.ppat.1002480) 22291590 PMC3266939

[55] Fujihara K, Itoyama Y, Yu F, Kubo C, Goto I 1991 Cellular immune surveillance against HTLV-I infected T lymphocytes in HTLV-I associated myelopathy/tropical spastic paraparesis (HAM/TSP). J. Neurol. Sci. 105, 99–107. (10.1016/0022-510X(91)90125-Q) 1795176

[56] Saito M et al. 2003 Low frequency of CD94/NKG2A^+^ T lymphocytes in patients with HTLV-1-associated myelopathy/tropical spastic paraparesis, but not in asymptomatic carriers. Blood 102, 577–584. (10.1182/blood-2002-09-2855) 12560226

[57] Yu F, Itoyama Y, Fujihara K, Goto I 1991 Natural killer (NK) cells in HTLV-I-associated myelopathy/tropical spastic paraparesis – decrease in NK cell subset populations and activity in HTLV-I seropositive individuals. J. Neuroimmunol. 33, 121–128. (10.1016/0165-5728(91)90056-D) 2066395

[58] Jeffery KJ et al. 1999 HLA alleles determine human T-lymphotropic virus-I (HTLV-I) proviral load and the risk of HTLV-I-associated myelopathy. Proc. Natl Acad. Sci. USA 96, 3848–3853. (10.1073/pnas.96.7.3848) 10097126 PMC22383

[59] Seich al Basatena NK et al. 2011 KIR2DL2 enhances protective and detrimental HLA class I-mediated immunity in chronic viral infection. PLoS Pathog. 7, e1002270 (10.1371/journal.ppat.1002270) 22022261 PMC3192839

[60] Iwasaki Y, Ohara Y, Kobayashi I, Akizuki S 1992 Infiltration of helper/inducer T lymphocytes heralds central nervous system damage in human T-cell leukemia virus infection. Am. J. Pathol. 140, 1003–1008. 1374584 PMC1886515

[61] Goon PK, Hanon E, Igakura T, Tanaka Y, Weber JN, Taylor GP, Bangham CR 2002 High frequencies of Th1-type CD4^+^ T cells specific to HTLV-1 Env and Tax proteins in patients with HTLV-1-associated myelopathy/tropical spastic paraparesis. Blood 99, 3335–3341. (10.1182/blood.V99.9.3335) 11964301

[62] Toulza F, Nosaka K, Tanaka Y, Schioppa T, Balkwill F, Taylor GP, Bangham CR 2010 Human T-lymphotropic virus type 1-induced CC chemokine ligand 22 maintains a high frequency of functional FoxP3^+^ regulatory T cells. J. Immunol. 185, 183–189. (10.4049/jimmunol.0903846) 20525891 PMC3575032

[63] Toulza F, Heaps A, Tanaka Y, Taylor GP, Bangham CR 2008 High frequency of CD4^+^FoxP3^+^ cells in HTLV-1 infection: inverse correlation with HTLV-1-specific CTL response. Blood 111, 5047–5053. (10.1182/blood-2007-10-118539) 18094326 PMC2602587

[64] Bangham CR, Toulza F 2011 Adult T cell leukemia/lymphoma: FoxP3^+^ cells and the cell-mediated immune response to HTLV-1. Adv. Cancer Res. 111, 163–182. (10.1016/B978-0-12-385524-4.00004-0) 21704832

[65] Toulza F, Nosaka K, Takiguchi M, Pagliuca T, Mitsuya H, Tanaka Y, Taylor GP, Bangham CR 2009 FoxP3^+^ regulatory T cells are distinct from leukemia cells in HTLV-1-associated adult T-cell leukemia. Int. J. Cancer 125, 2375–2382. (10.1002/ijc.24664) 19544530

[66] Wattel E, Vartanian JP, Pannetier C, Wain-Hobson S 1995 Clonal expansion of human T-cell leukemia virus type I-infected cells in asymptomatic and symptomatic carriers without malignancy. J. Virol. 69, 2863–2868. 7707509 10.1128/jvi.69.5.2863-2868.1995 PMC188982

[67] Etoh K, Tamiya S, Yamaguchi K, Okayama A, Tsubouchi H, Ideta T, Mueller N, Takatsuki K, Matsuoka M 1997 Persistent clonal proliferation of human T-lymphotropic virus type I-infected cells *in vivo*. Cancer Res. 57, 4862–4867. 9354450

[68] Taylor GP et al. 2006 Zidovudine plus lamivudine in human T-Lymphotropic virus type-I-associated myelopathy: a randomised trial. Retrovirology 3, 63 (10.1186/1742-4690-3-63) 16984654 PMC1590049

[69] Tanaka-Nakanishi A, Yasunaga J, Takai K, Matsuoka M 2014 HTLV-1 bZIP factor suppresses apoptosis by attenuating the function of FoxO3a and altering its localization. Cancer Res. 74, 188–200. (10.1158/0008-5472.CAN-13-0436) 24177179

[70] Kawatsuki A, Yasunaga JI, Mitobe Y, Green PL, Matsuoka M 2016 HTLV-1 bZIP factor protein targets the Rb/E2F-1 pathway to promote proliferation and apoptosis of primary CD4^+^ T cells. Oncogene 35, 4509–4517. (10.1038/onc.2015.510) 26804169 PMC4959989

[71] Yasuma K, Yasunaga J, Takemoto K, Sugata K, Mitobe Y, Takenouchi N, Nakagawa M, Suzuki Y, Matsuoka M 2016 HTLV-1 bZIP factor impairs anti-viral immunity by inducing co-inhibitory molecule, T cell immunoglobulin and ITIM domain (TIGIT). PLoS Pathog. 12, e1005372 (10.1371/journal.ppat.1005372) 26735971 PMC4703212

[72] Sugata K et al. 2016 HTLV-1 viral factor HBZ induces CCR4 to promote T-cell migration and proliferation. Cancer Res. 76, 5068–5079. (10.1158/0008-5472.CAN-16-0361) 27402079

[73] Satou Y, Yasunaga J, Yoshida M, Matsuoka M 2006 HTLV-I basic leucine zipper factor gene mRNA supports proliferation of adult T cell leukemia cells. Proc. Natl Acad. Sci. USA 103, 720–725. (10.1073/pnas.0507631103) 16407133 PMC1334651

[74] Mitobe Y, Yasunaga J, Furuta R, Matsuoka M 2015 HTLV-1 bZIP factor RNA and protein impart distinct functions on T-cell proliferation and survival. Cancer Res. 75, 4143–4152. (10.1158/0008-5472.CAN-15-0942) 26383166

[75] Okamoto K, Fujisawa J, Reth M, Yonehara S 2006 Human T-cell leukemia virus type-I oncoprotein Tax inhibits Fas-mediated apoptosis by inducing cellular FLIP through activation of NF-κB. Genes Cells 11, 177–191. (10.1111/j.1365-2443.2006.00927.x) 16436054

[76] Krueger A et al. 2006 HTLV-1 Tax protects against CD95-mediated apoptosis by induction of the cellular FLICE-inhibitory protein (c-FLIP). Blood 107, 3933–3939. (10.1182/blood-2005-06-2567) 16403915

[77] Iwanaga R, Ohtani K, Hayashi T, Nakamura M 2001 Molecular mechanism of cell cycle progression induced by the oncogene product Tax of human T-cell leukemia virus type I. Oncogene 20, 2055–2067. (10.1038/sj.onc.1204304) 11360190

[78] Peloponese JM Jr, Jeang KT 2006 Role for Akt/protein kinase B and activator protein-1 in cellular proliferation induced by the human T-cell leukemia virus type 1 Tax oncoprotein. J. Biol. Chem. 281, 8927–8938. (10.1074/jbc.M510598200) 16436385

[79] Ma G, Yasunaga J, Akari H, Matsuoka M 2015 TCF1 and LEF1 act as T-cell intrinsic HTLV-1 antagonists by targeting Tax. Proc. Natl Acad. Sci. USA 112, 2216–2221. (10.1073/pnas.1419198112) 25646419 PMC4343127

[80] Taniguchi Y, Nosaka K, Yasunaga J, Maeda M, Mueller N, Okayama A, Matsuoka M 2005 Silencing of human T-cell leukemia virus type I gene transcription by epigenetic mechanisms. Retrovirology 2, 64 (10.1186/1742-4690-2-64) 16242045 PMC1289293

[81] Satou Y et al. 2016 The retrovirus HTLV-1 inserts an ectopic CTCF-binding site into the human genome. Proc. Natl Acad. Sci. USA 113, 3054–3059. (10.1073/pnas.1423199113) 26929370 PMC4801255

[82] Furukawa Y, Fujisawa J, Osame M, Toita M, Sonoda S, Kubota R, Ijichi S, Yoshida M 1992 Frequent clonal proliferation of human T-cell leukemia virus type 1 (HTLV-1)-infected T cells in HTLV-1-associated myelopathy (HAM-TSP). Blood 80, 1012–1016. 1498321

[83] Kitze B, Usuku K 2002 HTLV-1-mediated immunopathological CNS disease. Curr. Top. Microbiol. Immunol. 265, 197–211. (10.1007/978-3-662-09525-6_10) 12014190

[84] Kataoka K et al. 2015 Integrated molecular analysis of adult T cell leukemia/lymphoma. Nat. Genet. 47, 1304–1315. (10.1038/ng.3415) 26437031

[85] Nakagawa M, Schmitz R, Xiao W, Goldman CK, Xu W, Yang Y, Yu X, Waldmann TA, Staudt LM 2014 Gain-of-function CCR4 mutations in adult T cell leukemia/lymphoma. J. Exp. Med. 211, 2497–2505. (10.1084/jem.20140987) 25488980 PMC4267233

[86] Nishimura S, Asou N, Suzushima H, Okubo T, Fujimoto T, Osato M, Yamasaki H, Lisha L, Takatsuki K 1995 p53 gene mutation and loss of heterozygosity are associated with increased risk of disease progression in adult T cell leukemia. Leukemia 9, 598–604. 7723391

[87] Pancewicz J, Taylor JM, Datta A, Baydoun HH, Waldmann TA, Hermine O, Nicot C 2010 Notch signaling contributes to proliferation and tumor formation of human T-cell leukemia virus type 1-associated adult T-cell leukemia. Proc. Natl Acad. Sci. USA 107, 16 619–16 624. (10.1073/pnas.1010722107) 20823234 PMC2944748

[88] Cook LB, Melamed A, Niederer H, Valganon M, Laydon D, Foroni L, Taylor GP, Matsuoka M, Bangham CR 2014 The role of HTLV-1 clonality, proviral structure, and genomic integration site in adult T-cell leukemia/lymphoma. Blood 123, 3925–3931. (10.1182/blood-2014-02-553602) 24735963 PMC4064332

[89] Laydon DJ et al. 2014 Quantification of HTLV-1 clonality and TCR diversity. PLoS Comput. Biol. 10, e1003646 (10.1371/journal.pcbi.1003646) 24945836 PMC4063693

[90] Tomasetti C, Vogelstein B 2015 Cancer etiology. Variation in cancer risk among tissues can be explained by the number of stem cell divisions. Science 347, 78–81. (10.1126/science.1260825) 25554788 PMC4446723

[91] Grassmann R, Dengler C, Muller-Fleckenstein I, Fleckenstein B, McGuire K, Dokhelar MC, Sodroski JG, Haseltine WA 1989 Transformation to continuous growth of primary human T lymphocytes by human T-cell leukemia virus type I X-region genes transduced by a Herpesvirus saimiri vector. Proc. Natl Acad. Sci. USA 86, 3351–3355. (10.1073/pnas.86.9.3351) 2541443 PMC287130

[92] Akagi T, Ono H, Shimotohno K 1995 Characterization of T cells immortalized by Tax1 of human T-cell leukemia virus type 1. Blood 86, 4243–4249. 7492783

[93] Grossman WJ, Kimata JT, Wong FH, Zutter M, Ley TJ, Ratner L 1995 Development of leukemia in mice transgenic for the *tax* gene of human T-cell leukemia virus type I. Proc. Natl Acad. Sci. USA 92, 1057–1061. (10.1073/pnas.92.4.1057) 7862633 PMC42636

[94] Shikishima H, Ikeda H, Yamada S, Yamazaki H, Kikuchi K, Wakisaka A, Kasai N, Shimotono K, Yoshiki T 1997 HTLV-I pX transgenic rats: development of cytokine-producing mammary carcinomas and establishment of the pX mammary carcinoma cell lines. Leukemia 11(Suppl. 3), 70–72. 9209302

[95] Koiwa T, Hamano-Usami A, Ishida T, Okayama A, Yamaguchi K, Kamihira S, Watanabe T 2002 5′-Long terminal repeat-selective CpG methylation of latent human T-cell leukemia virus type 1 provirus *in vitro* and *in vivo*. J. Virol. 76, 9389–9397. (10.1128/JVI.76.18.9389-9397.2002) 12186921 PMC136445

[96] Miyazaki M, Yasunaga J, Taniguchi Y, Tamiya S, Nakahata T, Matsuoka M 2007 Preferential selection of human T-cell leukemia virus type 1 provirus lacking the 5′ long terminal repeat during oncogenesis. J. Virol. 81, 5714–5723. (10.1128/JVI.02511-06) 17344291 PMC1900290

[97] Yamagishi M et al. 2012 Polycomb-mediated loss of miR-31 activates NIK-dependent NF-κB pathway in adult T cell leukemia and other cancers. Cancer Cell 21, 121–135. (10.1016/j.ccr.2011.12.015) 22264793

[98] Shimoyama M 1991 Diagnostic criteria and classification of clinical subtypes of adult T-cell leukaemia-lymphoma. A report from the Lymphoma Study Group (1984–87). Br. J. Haematol. 79, 428–437. (10.1111/j.1365-2141.1991.tb08051.x) 1751370

[99] Utsunomiya A et al. 2001 Improved outcome of adult T cell leukemia/lymphoma with allogeneic hematopoietic stem cell transplantation. Bone Marrow Transplant. 27, 15–20. (10.1038/sj.bmt.1702731) 11244433

[100] Okamura J et al. 2005 Allogeneic stem-cell transplantation with reduced conditioning intensity as a novel immunotherapy and antiviral therapy for adult T-cell leukemia/lymphoma. Blood 105, 4143–4145. (10.1182/blood-2004-11-4193) 15665110

[101] Bazarbachi A et al. 2010 Meta-analysis on the use of zidovudine and interferon-alfa in adult T-cell leukemia/lymphoma showing improved survival in the leukemic subtypes. J. Clin. Oncol. 28, 4177–4183. (10.1200/JCO.2010.28.0669) 20585095

[102] Taylor GP, Matsuoka M 2005 Natural history of adult T-cell leukemia/lymphoma and approaches to therapy. Oncogene 24, 6047–6057. (10.1038/sj.onc.1208979) 16155611

[103] Kchour G et al. 2009 Phase 2 study of the efficacy and safety of the combination of arsenic trioxide, interferon alpha, and zidovudine in newly diagnosed chronic adult T-cell leukemia/lymphoma (ATL). Blood 113, 6528–6532. (10.1182/blood-2009-03-211821) 19411628

[104] Yamano Y et al. 2009 Abnormally high levels of virus-infected IFN-γ^+^ CCR4^+^ CD4^+^ CD25^+^ T cells in a retrovirus-associated neuroinflammatory disorder. PLoS ONE 4, e6517 (10.1371/journal.pone.0006517) 19654865 PMC2715877

[105] Yamamoto K et al. 2010 Phase I study of KW-0761, a defucosylated humanized anti-CCR4 antibody, in relapsed patients with adult T-cell leukemia-lymphoma and peripheral T-cell lymphoma. J. Clin. Oncol. 28, 1591–1598. (10.1200/JCO.2009.25.3575) 20177026

[106] Ishii T et al. 2010 Defucosylated humanized anti-CCR4 monoclonal antibody KW-0761 as a novel immunotherapeutic agent for adult T-cell leukemia/lymphoma. Clin. Cancer Res. 16, 1520–1531. (10.1158/1078-0432.CCR-09-2697) 20160057

[107] Sugiyama D et al. 2013 Anti-CCR4 mAb selectively depletes effector-type FoxP3^+^CD4^+^ regulatory T cells, evoking antitumor immune responses in humans. Proc. Natl Acad. Sci. USA 110, 17 945–17 950. (10.1073/pnas.1316796110) PMC3816454 24127572

[108] Sugata K et al. 2016 Enhancement of anti-STLV-1/HTLV-1 immune responses through multimodal effects of anti-CCR4 antibody. Sci. Rep. 6, 27150 (10.1038/srep27150) 27250643 PMC4890010

